# Situating language in a minimal social context: how seeing a picture of the speaker’s face affects language comprehension

**DOI:** 10.1093/scan/nsab009

**Published:** 2021-01-20

**Authors:** David Hernández-Gutiérrez, Francisco Muñoz, Jose Sánchez-García, Werner Sommer, Rasha Abdel Rahman, Pilar Casado, Laura Jiménez-Ortega, Javier Espuny, Sabela Fondevila, Manuel Martín-Loeches

**Affiliations:** Cognitive Neuroscience Section, Center UCM-ISCIII for Human Evolution and Behaviour, Madrid 28029, Spain; Cognitive Neuroscience Section, Center UCM-ISCIII for Human Evolution and Behaviour, Madrid 28029, Spain; Department of Psychobiology & Methods for the Behavioural Sciences, Complutense University of Madrid, Madrid 28040, Spain; Cognitive Neuroscience Section, Center UCM-ISCIII for Human Evolution and Behaviour, Madrid 28029, Spain; Department of Psychology, Humboldt Universität zu Berlin, Berlin 10117, Germany; Department of Psychology, Humboldt Universität zu Berlin, Berlin 10117, Germany; Cognitive Neuroscience Section, Center UCM-ISCIII for Human Evolution and Behaviour, Madrid 28029, Spain; Department of Psychobiology & Methods for the Behavioural Sciences, Complutense University of Madrid, Madrid 28040, Spain; Cognitive Neuroscience Section, Center UCM-ISCIII for Human Evolution and Behaviour, Madrid 28029, Spain; Department of Psychobiology & Methods for the Behavioural Sciences, Complutense University of Madrid, Madrid 28040, Spain; Cognitive Neuroscience Section, Center UCM-ISCIII for Human Evolution and Behaviour, Madrid 28029, Spain; Cognitive Neuroscience Section, Center UCM-ISCIII for Human Evolution and Behaviour, Madrid 28029, Spain; Department of Psychobiology & Methods for the Behavioural Sciences, Complutense University of Madrid, Madrid 28040, Spain; Cognitive Neuroscience Section, Center UCM-ISCIII for Human Evolution and Behaviour, Madrid 28029, Spain; Department of Psychobiology & Methods for the Behavioural Sciences, Complutense University of Madrid, Madrid 28040, Spain

**Keywords:** situated language, multimodal, EEG, semantics, syntax

## Abstract

Natural use of language involves at least two individuals. Some studies have focused on the interaction between senders in communicative situations and how the knowledge about the speaker can bias language comprehension. However, the mere effect of a face as a social context on language processing remains unknown. In the present study, we used event-related potentials to investigate the semantic and morphosyntactic processing of speech in the presence of a photographic portrait of the speaker. In Experiment 1, we show that the N400, a component related to semantic comprehension, increased its amplitude when processed within this minimal social context compared to a scrambled face control condition. Hence, the semantic neural processing of speech is sensitive to the concomitant perception of a picture of the speaker’s face, even if irrelevant to the content of the sentences. Moreover, a late posterior negativity effect was found to the presentation of the speaker’s face compared to control stimuli. In contrast, in Experiment 2, we found that morphosyntactic processing, as reflected in left anterior negativity and P600 effects, is not notably affected by the presence of the speaker’s portrait. Overall, the present findings suggest that the mere presence of the speaker’s image seems to trigger a minimal communicative context, increasing processing resources for language comprehension at the semantic level.

## 1. Introduction

Language is social by nature. During interpersonal communication, interlocutors usually face each other and there is a multimodal exchange of information. Most (neuro)linguistic studies disregarded visual contributions of social stimuli on linguistic comprehension (Dale *et al.*, 2013; Munster and Knoeferle, 2018). However, the number of studies on social factors affecting language processing is rapidly increasing (e.g. Dale *et al.*, 2013; Knoeferle, 2016; Rohr and Abdel Rahman, 2018). Following this line of research, the present study uses electrophysiological measures to investigate whether processing in the semantic and syntactic domains in connected speech is affected already by minimal visuo-social information, that is, a mere picture of the speaker’s face.

In face-to-face communication, language can be described as a multimodal process involving both the linguistic information contained in the auditory stream and the visual input received from the speaker’s facial speech movements, gaze or facial expressions (van Wassenhove, 2013; Peelle and Sommers, 2015; Rohr and Abdel Rahman, 2015; Hernández-Gutiérrez *et al.*, 2018). Auditory and visual streams converge into a situational model of language comprehension (Hagoort, 2017). In this context, two critical cues can be extracted from a face. On the one hand, a linguistic-related stream emerges from dynamic articulatory mouth movements, typically known as visual speech (Buchwald *et al.*, 2009; Crosse *et al.*, 2015). On the other hand, face-to-face encounters provide *static* (invariant) social information about the speaker (e.g. gender, age, identity), as well as *dynamic* social information via changeable attributes like gaze direction or facial expressions (Haxby *et al.*, 2000). In this sense, mentalizing is critical in human communication, enabling the listener to decode mental states and communicative intentions of the speaker based on the perception of his/her face (Kampe *et al.*, 2003; Senju and Johnson, 2009; Myllyneva and Hietanen, 2015). Moreover, faces convey powerful social cues related to the attentional focus (Bindemann *et al.*, 2007; Langton *et al.*, 2008). In this regard, in face-to-face interactions, special attention is payed to the speaker’s face (Vertegaal *et al.*, 2001), and at the same time, the speaker facing the listener enhances the feeling of being attended.

Behavioural studies have found that facial features, particularly the speaker’s gaze, facilitate language comprehension at lexico-semantic (Richardson and Dale, 2005; Holler *et al.*, 2014) or syntactic levels, for example, referent disambiguation (Hanna and Brennan, 2007), thematic role assignment, and syntactic structuring (Knoeferle and Kreysa, 2012). While there is extensive behavioural and neuroimaging evidence of visual integration of the speaker’s face in communicative situations, results from online comprehension measures are scarce. A fine-grained study in the temporal domain may be achieved with event-related potentials (ERP). Well-known ERP language–related indices are the N400, the left anterior negativity (LAN), and the P600 components of the ERP, explained below.

The N400 is a typical response to a meaningful stimulus, and its amplitude to a word in a sentence is related to its congruency with preceding information (Payne *et al.*, 2015), being therefore sensitive to contextual constraints (Kutas and Federmeier, 2011). This ERP component peaks around 400 ms, and its topography varies from centro-parietal to posterior sites (Van Petten, 2014; Schöne *et al.*, 2018). Recently, it has been also interpreted as a late mismatch negativity (MMN) reflecting precision-weighted prediction errors (Bornkessel-Schlesewsky and Schlesewsky, 2019). The LAN has been proposed to reflect first-pass syntactic processes (Molinaro *et al.*, 2011). This component peaks around 300 ms after the presentation of a morphosyntactic mismatch, and its amplitude is typically seen to indicate the difficulty of morphosyntactic integration (Friederici, 2002). The P600 is a positive component peaking around 600 ms or later after stimulus onset. The syntactic view of this component suggests that it reflects late syntactic analysis, repair, or reanalysis (Friederici, 2011; Brouwer *et al.*, 2012); however, the P600 has been also related to semantic manipulations, although with smaller amplitudes than syntactic mismatches. The semantic P600 emerges, for example, to semantic reversal anomalies (‘*The hearty meal was devouring the kids’* in comparison to ‘*The hearty meal was devoured by the kids’*) (Kolk *et al.*, 2003; Vissers *et al.*, 2007) or to words that are semantically incongruent to the embedding sentence (Roehm *et al.*, 2007; Van Petten and Luka, 2012). A recent domain-general interpretation considers the P600 as a variant of P300 (Sassenhagen *et al.*, 2014). Accordingly, instead of reflecting structural or combinatorial operations, the P600 would relate to recognition and categorization processes, and its amplitude would reflect the salience or significance of the stimulus category. P600 effects would therefore be stronger or more likely to occur after morphosyntactic anomalies than after semantic violations because the former are more categorical (Coulson *et al.*, 1998; Sassenhagen *et al.*, 2014). Indeed, similar components have been described in the P600 latency range that share certain characteristics, these being the P3b, the syntactic and semantic P600 and the late positive component, along with related components (e.g. late posterior positivity, LPP) (Leckey and Federmeier, 2020). Leaving aside domain-based explanations of these positivities, in addition to waveform similarities, they share a characteristic enhancement to unexpected stimuli. Accordingly, they seem functionally related to re-processing of information. At these late latencies, there are also other higher-order ERPs with negative polarity, like the late posterior negativity (LPN), related to processes endorsing information retrieval (Mecklinger *et al.*, 2016) and action monitoring (Johansson and Mecklinger, 2003). As mentioned above, although it is possible to characterize ERP components depending on the field of study, it may be more useful to consider the cognitive processes underlying these brain signatures than referring to different components in the basis of certain human behaviours (Donchin, 1981).

Previous electrophysiological research demonstrated that during language comprehension, listeners take into consideration the social context and their knowledge about the speaker (Van Berkum *et al.*, 2008; Bornkessel-Schlesewsky *et al.*, 2013; Jouralev *et al.*, 2018). Thus, an N400 effect has been reported to inconsistencies between voice characteristics and message content, for example when the sentence ‘Every evening I drink some *wine* before I go to sleep’ is uttered with a child’s voice (Van Berkum *et al.*, 2008). In another study, larger N400 effects were found to false political statements when the speaker was a politician compared to other speakers, but no difference was found for non-political statements (Bornkessel-Schlesewsky *et al.*, 2013). Moreover, language processing is also affected by the communicative context, which can be triggered by the belief that a real person is communicating through a computer (Schindler *et al.*, 2015; Schindler and Kissler, 2016, 2017). This communicative effect was reflected in an increased LPP to emotional words uttered by a real person rather than an artificial sender and was interpreted as an increase of motivated attention. In a previous study, we found a similar effect in an audiovisual context when congruent sentences were accompanied by a video of the speaker as compared to his static image (Hernández-Gutiérrez *et al.*, 2018). In the present study, we will focus on whether a static face is sufficient to elicit social effects on language processing. Hence, we will study language in a *minimal social context*.

The present study assessed the relevance of the presence of a speaker’s face in language comprehension. For this purpose, we situated spoken language within a simulated face-to-face context and investigated the influence of merely seeing the speaker’s image, displaying a direct gaze towards the listener, on real-time sentence processing as reflected in ERPs. Therefore, in this minimal and simulated social context, the manipulation concerns the physical aspect of the social context and does not include beliefs or attributions, since no other information about the speakers was given to the participants. In separate experiments, we manipulated the morphosyntactic agreement and semantic congruency of target words embedded in naturally spoken sentences. Participants listened to the sentences while viewing an image of the putative speaker or—as a control—a scrambled face. In Experiment 1, investigating semantic effects, we focussed on the N400 (and P600, if present), whereas in Experiment 2, we manipulated syntactic processing, exploring effects on LAN and P600. To date, no study has investigated the impact of the mere presence of the speaker’s face on speech comprehension with online measures.

Within this minimal social context, we expected increased attention to the linguistic message when the speaker’s face is perceived (relative to scrambled control stimuli) because of its relationship to and relevance for the communicative process. Attention to words can affect semantic processing and has been found to enhance the semantic N400 effect (McCarthy and Nobre, 1993). Further, since the semantic N400 is sensitive to context effects, we expected larger N400 effects when the face of the speaker is seen, providing a communicative context and, hence, inducing the allocation of additional attentional resources to the linguistic stream. It is likely that situating language in a minimal social context leads to a deeper linguistic processing due to self-referential mechanisms and perceived communicative intent (Wang *et al.*, 2011), since a visible person might be experienced as (intentionally) speaking to the participant. This manipulation may therefore induce deeper processing (Craik and Lockhart, 1972; Wang *et al.*, 2011) facilitating more detailed linguistic analysis, which might enhance the semantic N400 for spoken sentences (Wang *et al.*, 2011).

Conversely, syntactic processing has traditionally been considered as informationally encapsulated and consequently insensitive to non-linguistic stimuli (Friederici, 2002, Hauser *et al.*, 2002). However, there are numerous ERP findings questioning this claim (Martín-Loeches *et al.*, 2012; Casado *et al.*, 2018). Further, there is behavioural evidence on the relationship between the perception of gaze and syntactic processing (Hanna and Brennan, 2007; Knoeferle and Kreysa, 2012). Therefore, increased attention and deeper processing might occur also at the syntactic level. We therefore predicted that also the LAN and P600 effects may be enhanced by the minimal social context manipulation employed here.

## 2. Experiment 1. Semantic domain

### 2.1. Methods

#### 2.1.1. Participants.

Twenty-eight native Spanish speakers participated in this experiment (14 females; age range 18–24; *M* = 21.3 years). They were all right-handed (Mean Oldfield scores: +75) and declared normal or corrected to normal vision and hearing and absence of neurological disorders. Participants gave written informed consent and were reimbursed for participating in the experiment. The study was performed in accordance with the Declaration of Helsinki.

#### 2.1.2. Materials and procedure.

The language stimuli consisted of 480 sentences in Spanish with three different structures. In addition, a semantically incongruent version of each sentence was created. To this aim, critical words were pseudo-randomly shuffled between sentences. Depending on the structure of the sentence, the critical word could be an adjective (Structure 1) or a noun (Structures 2 and 3). In written stimuli, this semantic manipulation has been typically shown to elicit N400 effects (e.g. Martín-Loeches *et al.*, 2012). A congruent and incongruent example of each type of sentence is given in the Supplementary Material, Appendix 1.

**Fig. 1.**
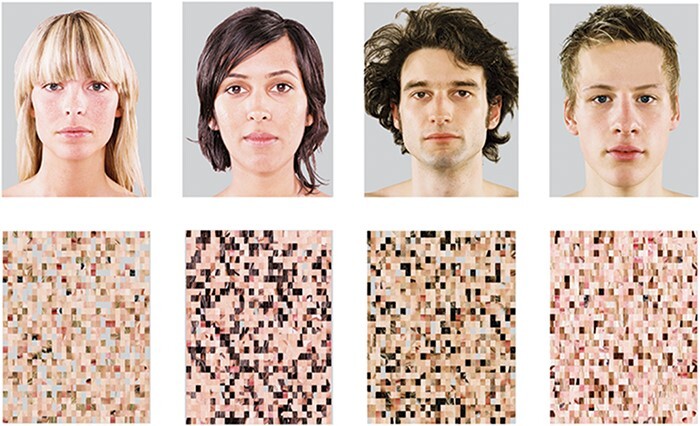
Visual stimuli. Pictures of the speakers (top) and their scrambled versions (bottom).

Every spoken sentence was accompanied by the presentation of either a static picture of the speaker’s face (social condition) or a scrambled face (control condition) (Figure 1). Two male and two female faces were taken from our own database. The scrambled version of the faces was created with Matlab Software using a 30 × 40 matrix. Therefore, the control stimuli kept most physical characteristics intact, but no facial features were identifiable. Each face was assigned to one of four voices (of matching sex), and the correspondence was kept consistent throughout the experiment. For a detailed explanation of the materials and procedure, see Supplementary material, Appendix 2.

Participants were informed that they would hear sentences while seeing the face of the speakers and should decide after each sentence whether it made sense by pressing one of two buttons on a response box. The button-decision assignments as well as the response hand were counterbalanced across participants. At the beginning of each trial, the speaker’s face appeared in the center of the screen, while the audio presentation started 500 ms later. The face was present until the sentence had ended. After 1 s, a question mark appeared at the center of the screen, prompting the response.

#### 2.1.3. EEG recording and data analysis.

The EEG was recorded from 59 electrodes placed within an elastic cap according to the international 10–20 system. Impedances were kept below 5 kΩ. Bipolar vertical and horizontal electrooculograms (HEOG and VEOG) were recorded with electrodes placed at the outer canthus of the eyes (HEOG) and below and above the right eye (VEOG). One electrode each was placed on the mastoids; the right mastoid served as initial reference. Signals were amplified with a band-pass from 0.01 to 100 Hz and sampled at 250 Hz.

EEG data were analyzed with Brain Vision Analyzer software. The EEG was re-referenced offline to average mastoids and an 8th-order, zero-phase, Butterworth filter was applied with a band-pass from 0.1 to 15 Hz. EEG epochs of 1600 ms were segmented from the continuous EEG data, starting 200 ms before the onset of the critical words. The correction of blink and horizontal electro-ocular artifacts was performed by means of Independent Component Analysis (ICA) as implemented in Brain Vision Analyzer®. The rejection of the remaining artifacts was performed semiautomatically, removing trials with activities exceeding a range of 100 µV. Overall, the mean rate of rejected trials was 13%. ERPs were computed only for trials followed by correct behavioral responses.

**Fig. 2.**
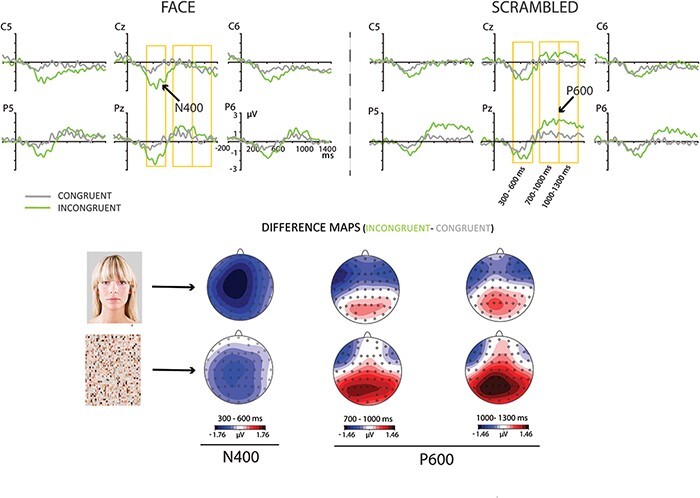
Effects of congruency for the speaker’s FACE and the SCRAMBLED face condition. The difference maps represent the congruency effects (INCONGRUENT minus CONGRUENT) for each visual condition.

Visual inspection of ERPs confirmed the expected central N400 effect and a parietal P600 effect (Figure 2). Therefore, repeated-measures ANOVAs were performed using two regions of interest (ROI). A central ROI for the N400 effect included electrode sites FC1, FCz, FC2, C1, Cz, C2, CP1, CPz and CP2. A parietal ROI for the P600 effect comprised electrodes P3, P1, Pz, P2, P4, PO3, PO4, CP1, CPz and CP2. Both ROIs included the collapsed values of the individual electrodes. The ANOVAs included factors Semantic Congruency (congruent vs incongruent) and Face Presence (face vs scrambled face). Based upon visual inspection of both the different waveforms and the topographies of the effect, ANOVAs included the following time windows after the critical word onset: 300–600 ms for the N400; 700–1000, and 1000–1300 ms for the P600. Bonferroni-corrected post hoc pairwise comparisons were applied to significant interactions. and Greenhouse-Geisser corrections were applied when appropriate.

A 200-ms post-stimulus baseline was applied. This sort of baseline has been previously used by others (e.g. Camblin *et al.*, 2007; Hutzler *et al.*, 2007; Lau *et al.*, 2016). Our target words were located within connected-speech sentences, and the ERPs in the congruent and incongruent conditions diverged before the onsets of the critical word (for a similar explanation, see Camblin *et al.*, 2007). Therefore, a standard prestimulus baseline (e.g. −200 ms) would not have fully captured the effects of the experimental manipulations on the N400 and P600 amplitudes. By using a post-stimulus baseline, we can be more confident that the differences found between conditions are related to the experimental manipulations.

### 2.2. Results and discussion

The mean error rate in the semantic congruency task was 11.38%. An ANOVA including the repeated measures factors Semantic Congruency and Face Presence revealed a significant effect of Semantic Congruency (*F* (1,27) = 10.85; *P* = 0.002; *η^2^* = 0.26). Incongruent sentences showed higher error rate (14.98%) than congruent sentences (7.82%). Neither Face Presence nor the interaction of Semantic Congruency by Face Presence yielded significant results (*F*’s < 1; *P*’*s* > 0.05; *η^2^* < 0.01).

Regarding reaction times, ANOVA revealed a significant main effect of Semantic Congruency (*F* (1,27) = 4.78; *P* = 0.029; *η^2^* = 0.003). Congruent sentences (*M* = 366.0 ms) were associated with shorter reactions times compared to incongruent sentences (*M* = 379.9 ms). Factor Face Presence was not significant (*F* (1,27) = 2.47; *P* = 0.12; *η^2^* = 0.002), although reaction times for the face condition were shorter (M = 367.2 ms) than those for the scrambled face (M = 377.0 ms). The interaction of Semantic Congruency and Face Presence was not significant *(F* (1,27) = 1.15; *P* = 0.28; *η^2^* = 0.001).

Figure 2 shows grand-average ERPs to congruent and incongruent words at six selected electrodes. The N400 effect exhibits a broad scalp distribution, which may be related to the use of spoken language (e.g. Li *et al.*, 2008; Wang *et al.*, 2011). The ANOVA computed in the N400 time window yielded a significant effect of Congruency (*F* (1,27) = 20.67; *P* < 0.001; *η^2^* = 0.43). Face Presence did not yield a significant main effect in this interval (*F* (1,27) = 1.57; *P* = 0.220; *η^2^* = 0.05). Importantly, Congruency by Face Presence showed a significant interaction (*F* (1,27) = 4.44; *P* = 0.044; *η^2^* = 0.14), with a larger N400 effect when facing the speaker than the control condition. Post hoc pairwise comparisons for this interval revealed that the N400 was significantly larger in the face condition than the scrambled face for incongruent sentences (Δ = 0.97 µV, *p* = 0.021). However, the difference was not significant for congruent sentences (Δ = 0.26 µV, *p* = 0.538).

**Fig. 3.**
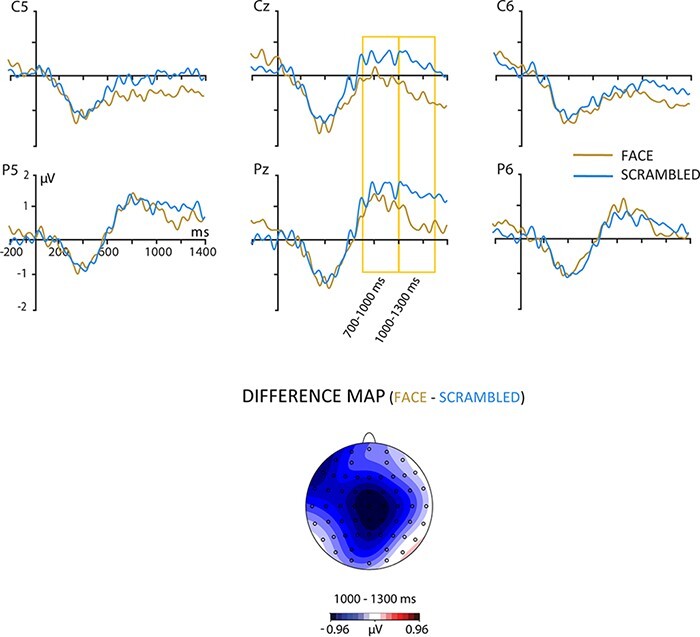
Main effect of face presence. The topography represents the late posterior negativity (LPN) effect (FACE minus SCRAMBLED face).

As to the P600, Congruency as a main effect was significant for both analyzed intervals (700–1000 ms: *F* (1,27) = 15.87; *P* < 0.001; *η^2^* = 0.37; and 1000–1300 ms: *F* (1,27) = 14.53; *P* = 0.001; *η^2^* = 0.35). In contrast to the N400, the interaction Congruency by Face Presence in the P600 effect did not reach statistical significance (700–1000 ms: *F* (1,27) = 1.02; *P* = 0.167; *η^2^* = 0.07; and 1000–1300 ms: *F* (1,27) = 2.62; *P* = 0.117; *η^2^* = 0.09). Interestingly, a main effect of Face Presence was significant between 700 and 1300 ms (700–1000 ms: *F* (1,27) = 5.0; *P* = 0.034; *η^2^* = 0.16; 1000–1300 ms: *F* (1,27) = 5.69; *P* = 0.024; *η^2^* = 0.17). Figure 3 shows the grand average ERP to the speaker’s face compared to the control condition. The effect exhibits a centro-parietal distribution with negative polarity that is larger to the speaker’s face.

Summarizing, in Experiment 1, the semantic N400 effect was sensitive to the type of visual stimuli presented. In line with our hypothesis, the N400 effect was larger when the auditory sentence was associated with a speaker’s face as compared to a scrambled image. This result could be explained by attentional processing effects because attention may be boosted by the presence of a putative speaker in the communicative visual context, which could impact semantic processing (McCarthy and Nobre, 1993; Van Petten, 2014), while mentalizing processes may increase the depth of processing (Wang *et al.*, 2011).

The main effect to the speaker’s face was unexpected. This negativity resembles an LPN effect (Johansson and Mecklinger, 2003; Mecklinger *et al.*, 2016). Results of Experiment 2 will be useful to better understand this effect and probe its consistency (see the *General discussion* section for a suggested explanation of this ERP modulation). Experiment 2 will also help to verify whether syntactic processing is sensible to this minimal social context manipulation.

## 3. Experiment 2. Syntactic domain

### 3.1. Methods

#### 3.1.1. Participants.

Participants were the same as in Experiment 1. The order of the experiments was counterbalanced; therefore, half of the participants completed Experiment 2 before Experiment 1.

#### 3.1.2. Materials and procedure.

The same 480 sentences used in Experiment 1 had been modified to include morphosyntactic violations of gender or number in critical words. Depending on the structure of the sentence, there could be a noun-adjective mismatch (Structure 1) or a determiner-noun mismatch (Structures 2 and 3). Both types of morphosyntactic errors typically elicit LAN and P600 components. An example of the sentences by type of structure is given in the Supplementary Material, Appendix 1. Participants listened to 240 sentences, different to the ones presented for a specific participant in Experiment 1, so that at the end of the whole study every participant had heard all 480 sentences once. For this experiment, given that the critical information for morphosyntactic violations relates to the gender/number markers, triggers were set on critical words at the offset of the lexeme, just before the gender/number declension. This was done following the same procedure as for the materials of Experiment 1 (average of 3 different judgements).

The procedure was similar to Experiment 1, but participants performed a morphosyntactic correctness task after the presentation of each sentence.

#### 3.1.3. EEG recording and data analysis.

The characteristics of the EEG recordings were identical to those in Experiment 1.

**Fig. 4.**
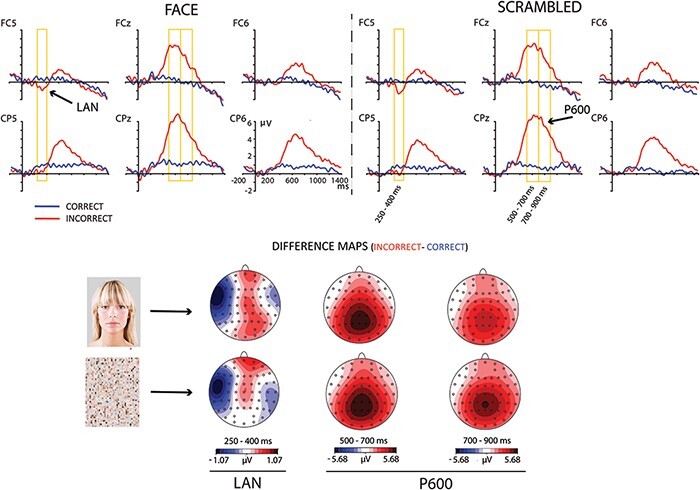
Effect of morphosyntactic correctness. ERPs are plotted separately for each visual context. The DIFFERENCE MAPS (INCORRECT minus CORRECT) represent the topographies of the LAN and the P600 effect.

Visual inspection of ERPs confirmed the LAN and P600 effects (Figure 4). In this Experiment, repeated-measures ANOVAs were also performed using two ROIs. An anterior left ROI for the LAN effect included electrode sites F7, F5, FT7, FC5, T7 and C5. A parietal ROI for the P600 effect comprised the same electrodes than Experiment 1: P3, P1, Pz, P2, P4, PO3, PO4, CP1, CPz and CP2. Both ROIs included the collapsed values of the individual electrodes. The data processing, except for component parametrization, was the same as for Experiment 1. Overall, mean rate of rejected epochs was 11%. The same type of ANOVA including factors Morphosyntactic Correctness and Face Presence was employed as for Experiment 1 and applied to the following time segments after morphosyntactic declension onset: 250–400 ms for the LAN; 500–700 and 700–900 ms for the P600. Since in Experiment 1 Face Presence had been significant between 1000 and 1300 ms, we performed an ANOVA at this time window in Experiment 2 as well.

#### 3.1.4. Results and discussion.

The mean error rate in the morphosyntactic correctness task was 5.39%. ANOVA revealed a significant main effect of Morphosyntactic Correctness (*F* (1,27) = 8.09; *P* = 0.008; *η^2^* = 0.231). Incorrect sentences were related to a higher mean error rate (6.26%) than correct ones (4.51%). The factor Face Presence (*F* (1,27) = 0.11; *P* = 0.74; *η^2^* = 0.004) and its interaction with Morphosyntactic Correctness (*F* (1,27) = 1.65; *P* = 0.209; *η^2^* = 0.06) were not significant.

The ANOVA for the reaction times revealed significant main effects of both Morphosyntactic Correctness (*F* (1,27) = 63.63; *P* < 0.001 *η^2^* = 0.04) and Face Presence (*F* (1,27) = 43.28; *P* < 0.001; *η^2^* = 0.02). Correct sentences (M = 379.03 ms) were associated with longer mean reaction times than incorrect sentences (M = 341.08 ms), and reaction times for sentences accompanied by the speaker’s face were significantly shorter (M = 343.53 ms) than the scrambled face condition (M = 377.31 ms). The interaction of Morphosyntactic Correctness and Face Presence was not significant (*F* (1,27) = 0.42; *P* = 0.84; *η^2^* < 0.000).

Figure 4 shows the grand average ERP waveforms. Morphosyntactic violations elicited a LAN distributed at left fronto-temporal sites between 200 and 450 ms, followed by a prominent centro-parietal P600 between 400 and 1100 ms. In the LAN interval, ANOVA revealed significant main effects of Morphosyntactic Correctness (*F* (1,27) = 18.59; *P* < 0.001; *η^2^* = 0.41). Face Presence and its interaction failed significance by a wide margin (*F*’s < 1; *p*’s > 0.46). Therefore, the LAN did not seem to be affected by the visual context.

Morphosyntactic violations also elicited a P600 component, which showed strong main effects of Morphosyntactic Correctness (500–700 ms: *F* (1,27) = 128.4; *P* < 0.001; *η^2^* = 0.83, and 700–900 ms: *F* (1,27) = 112.5; *P* < 0.001; *η^2^* = 0.81). In contrast to the P600 in Experiment 1, Face Presence as a main effect did not induce significant effects in the P600 interval (500–700 ms: *F* (1,27) = 0.76; *P* = 0.39; *η^2^* = 0.03; and 700–900 ms: *F* (1,27) = 0.60; *P* = 0.44; *η^2^* = 0.02). Importantly, the P600 effect was not significantly modulated by the speaker’s face. However, the interaction Face Presence by Morphosyntactic Correctness yielded a strong trend and almost reached statistical significance in the later interval of the component, being smaller in presence of the face (500–700: *F* (1,27) = 0.35; *P* = 0.85; *η^2^* = 0.00; 700–900 ms: *F* (1,27) = 3.9; *P* = 0.058; *η^2^* = 0.13).

Finally, the extended ANOVA exploring Face Presence in the 1000–1300 ms segment did not yield a significant main effect (*F* (1,27) = 0.55; *P* = 0.46; *η^2^* = 0.01). Therefore, we can be sure that this late effect is specific of the Semantic Experiment (Experiment 1).

Overall, morphosyntactic processing was not significantly affected by the minimal social context. The LAN effect showed almost identical amplitude and distribution in both visual contexts. On the other hand, the P600 effect was visually larger in the control condition, but statistical testing did not support a significant difference. Interestingly, there was no main effect of the speaker’s face in this experiment. This suggests that the negativity found in Experiment 1 to the speaker’s face is probably dependent of the semantic nature of the experimental task. This result will be addressed in the *General discussion* section.

## 4. General discussion

The current study aimed to investigate how the mere presence of a speaker’s static face may influence semantic and morphosyntactic processing of natural speech. We explored these effects by means of the language-related ERP modulations N400, LAN and P600. To this end, two separate experiments were performed. In spite of the problems associated to the use of spoken language stimuli, which implies incremental stimulus presentation, differences in word length and jittering of ERPs (Alday *et al.*, 2017), we obtained all the ERP components expected in the conditions created.

The presence of the speaker’s face appears to have a clear impact on semantic processing. Experiment 1 showed an interaction of visual context and semantic comprehension in the latency of the N400, with larger amplitudes when language was situated in a minimal social context. This finding is in accordance with our hypothesis and could be explained as a competition about the resources allocated to the face and the voice inputs. Considering that the amplitude of the N400 is positively related to the difficulty of semantic comprehension (e.g. Kutas and Federmeyer, 2011), it appears that it is necessary to invest more resources in the semantic comprehension of the incongruent sentences when both face and word stimuli are concomitantly processed. This result is in line with previous studies reporting larger N400 effects when attention is explicitly oriented to the linguistic stimuli (McCarthy and Nobre, 1993) or when the depth of processing is enhanced (Wang *et al.*, 2011). Accordingly, participants seem to attend more to the incongruent critical words when situated in a minimal communicative context. Our experiment simulates a face-to-face context and demonstrates that semantic comprehension is not only affected when participants receive information from the speaker that is semantically incongruent relative to the linguistic message—e.g. gaze shifts or previous knowledge about the speaker that are incongruent with the content of the sentences (Bornkessel-Schlesewsky *et al.*, 2013; Jachmann *et al.*, 2019) but also when the incoherent information is processed in presence of the speaker.

The effect of the speaker’s face may be due to the interplay of different factors. First, faces—and particularly the eyes—are unlike any other visual stimulus because of their capacity to capture larger amounts of attentional resources (Senju and Hasegawa, 2005; Langton *et al.*, 2008). Further, it can be argued that in a communicative context, the listener’s motivated attention to the speaker is increased (Schindler *et al.*, 2015; Schindler and Kissler, 2016, 2017; Hernández-Gutiérrez *et al.*, 2018), which in turn would lead to deeper processing of the message (Craik and Lockhart, 1972; Wang *et al.*, 2011). On the other hand, seeing a face with direct gaze—as was the case here—has been related to the activation of social brain circuits (Brunet *et al.*, 2000; Adams *et al.*, 2010), some of which also take part in the comprehension of language (e.g. Redcay, 2008; Cechini *et al.*, 2013). Therefore, the activation of social brain networks may strengthen attention to the linguistic message. At first sight, results of the present work may seem difficult to reconcile with those by Hernández-Gutiérrez *et al.* (2018), where the N400 was insensitive to facial dynamics. However, in that study, language processing was always situated in a social context, either dynamic or static. Accordingly, the social situational context (presence vs absence of the speaker’s portrait) may be more effective than the facial movements of the speaker’s face (dynamic vs static) in modulating the N400 semantic effect.

In contrast to the semantic N400 effects, the ERPs elicited by morphosyntactic manipulations, LAN or P600, were not significantly affected by the presence of the speaker’s face. It seems that the minimal social context manipulated here could not affect enough the neural processes involved in morphosyntactic processing. Speaker’s gaze, nevertheless, has been reported to interact with the processing of syntactic structures, as a function of the coordination between both the linguistic and the visual streams (Knoeferle and Kreysa, 2012). It is possible that our paradigm did not meet the requirements to yield a significant visual effect on syntactic processing. Seeing the speaker’s static face with direct gaze may not be a suitable visual stimulus to significantly influence the neural syntactic processes. Nonetheless, reaction times for sentences accompanied by the speaker’s face were significantly shorter than in the scrambled face condition in the second (syntactic) experiment. This finding indicates, interestingly, overall effects of the presence of a face in a syntactic task, even if the ERP fluctuations studied here have not been able to grasp them. In future studies, it would be interesting to manipulate gaze movements in this frame.

Regardless of the linguistic manipulation, a late negativity arose between 700 and 1300 ms in the presence of a face (Figure 3). Interestingly, this ERP modulation only emerged in Experiment 1, in which participants had to judge semantic congruency, contrasting with the morphosyntactic correctness task in Experiment 2. Both experiments had the same stimulation procedures as well as exactly the same visual and linguistic stimuli (with the exception of the type of errors in the incorrect versions of the sentences). This effect could be explained by differences in the main cognitive processes involved during each experiment. In this regard, the behavioral results evinced more difficulties to perform the semantic congruency task than the morphosyntactic task. Indeed, after the experiment, some participants stated that this difficulty was related to the possible metaphoric interpretation of several semantic incongruities, which hampered their decision. Therefore, semantic processing of incongruences appears more demanding, particularly because it relies upon memory processes (Kutas and Federmeier, 2011), while morphosyntactic correctness decision is a more categorical task, typically considered as automatic (Coulson *et al.*, 1998; Sassenhagen *et al.*, 2014). Considering the time course of the ERP modulation, its negative polarity, the centro-parietal distribution and its dependence on the experimental task, we interpret it as the LPN effect. This neurophysiological response is related to highly demanding processes, such as information retrieval (Mecklinger *et al.*, 2016), action monitoring (Johansson and Mecklinger, 2003), response-related processes in recognition memory tasks (Wilding and Rugg, 1997) or higher order stimulus evaluation (Sommer *et al.*, 2018). The LPN effect may therefore have emerged in our semantic task but not in the morphosyntactic one due to the higher levels of difficulty of the former, together with the higher complexity of the social context and the communicative situation when compared to the control (non-face) situation.

Summarizing, we have shown that an outstanding social variable, namely the presence of the speaker’s face, affects language comprehension at least in the semantic domain. This has been the case even if the social variable here explored is utterly irrelevant to the content of the linguistic message. These findings are of interest for future research studying language in face-to-face communication contexts and uphold the idea that language should be ideally studied in situations more realistic than the typically used, such as written language comprehension, or listening to sentences without a minimal social context.

## Supplementary Material

nsab009_Supp
