# Supplementary material for: Situating language in a minimal social context: how seeing a picture of the speaker’s face affects language comprehension
Source: Soc Cogn Affect Neurosci. 2021 Jan 20;16(5):502–11. doi: 10.1093/scan/nsab009 (PMC8094999; doi:10.1093/scan/nsab009)
Supplement: nsab009_Supp [file nsab009_supp.zip › SUPPLEMENTARY MATERIAL.docx]

**SUPPLEMENTARY MATERIAL**

*APPENDIX 1: LINGUISTIC STIMULI EXAMPLES*

Note: Critical words in bold. English translation in brackets. Please note that the noun-adjective order in Spanish is reversed in the English version.

1. *Experiment 1 (semantic manipulation)*

Structure 1 (n=300): [Det]-[N]-[**Adj**]-[V]-[Prep]-[N]

- Congruent sentence: *El pañuelo* ***bordado*** *era de mi abuela* (The **embroided** cushion belonged to my grandmother)
- Incongruent sentence: *El pañuelo* ***asado*** *era de mi abuela* (The **roasted** cushion belonged to my grandmother)

Structure 2 (n=90): [Det]-[N]- [V]-[Det]-[**N**]- [Adj]

- Congruent sentence: *Los turistas habían fotografiado los* ***glaciares*** *árticos*. (The tourists had photographed the arctic **glaciers**)
- Incongruent sentence*: Los turistas habían fotografiado los* ***pensamientos*** *árti*cos (The tourists had photographed the arctic **thoughts)**

Structure 3 (n=90): [Det]-[N]- [V] -[Prep]-[Det]-[**N**]-[Prep]-[Det]-[N]

- Congruent sentence: Las hojas son recogidas durante el **otoño** por los barrenderos (The leaves are picked by the sweepers during the **autumn**)
- Incongruent sentence: Las hojas son recogidas durante el **escritor** por los barrenderos. (The leaves are picked by the sweepers during the **writer**)

1. *Experiment 2 (morphosyntactic manipulation)*

Structure 1 (n = 300): [Det]-[N]-[**Adj**]-[V]-[Prep]-[N]

- Correct sentence: *El pañuelo_Masc/Sing_* ***bordado*** *_Masc/Sing_* *era de mi abuela* (The **embroided** *_Masc/Sing_* cushion *_Masc/Sing_* belonged to my grandmother)
- Incorrect sentence: *El pañuelo _Masc/Sing_* ***bordada*** *_Fem/Sing_ era de mi abuela* (The **embroided** *_Fem/Sing_* cushion *_Masc/Sing_* belonged to my grandmother)

Structure 2 (n = 90): [Det]-[N]- [V]-[Det]-[**N**]- [Adj]

- Correct sentence: *Los turistas habían fotografíado los _Masc/Plur_* ***glaciares*** *_Masc/Plur_ árticos*. (The tourists had photographed the *_Masc/Plur_* arctic **glaciers** *_Masc/Plur_*)
- Incorrect sentence*: Los turistas habían fotografiado los _Masc/Plur_* ***glaciar*** *_Masc/Sing_ árti*cos (The tourists had photographed the *_Masc/Plur_* arctic **glacier** *_Masc/Sing_***)**

Structure 3 (n=90): [Det]-[N]- [V] -[Prep]-[Det]-[**N**]-[Prep]-[Det]-[N]

- Correct sentence: *Las hojas son recogidas durante el _Masc/Sing_* ***otoño*** *_Masc/Sing_* *por los barrenderos* (The leaves are picked by the sweepers during the *_Masc/Sing_* **autumn** *_Masc/Sing_*)
- Incorrect sentence: *Las hojas son recogidas durante el _Masc/Sing_* ***otoños*** *_Masc/Plur_* *por los barrenderos*. (The leaves are picked by the sweepers during the *_Masc/Sing_* **autumns** *_Masc/Plur_*)

*APPENDIX 2: DETAILED MATERIALS AND PROCEDURE*

The whole set of 960 sentences (480 correct plus 480 incorrect versions) were spoken with neutral prosody by a female and a male speaker. One additional male and female voice was created by manipulating the fundamental frequency (F_0_) with Praat software. These voices were created by increasing the F_0_ of the male voice by 10% and reducing the F_0_ of the female voice by 10%. This manipulation resulted in two different male and female voices each, per sentence. We decided not to use four different speakers in order to minimize speech variability. All audio files were matched in intensity with Audacity software. Three independent researchers set the ERP triggers at the onset of each target word with GoldWave software considering the auditory and visual (spectrogram) patterns of the sound waves. Thereafter, the three triggers (one per judge) for a given word were averaged to obtain a single time-point for each word. This procedure has been successfully employed in previous work (Hernandez-Gutierrez et al., 2018). The length of target words varied between two and five syllables, and linguistic characteristics like word frequency, concreteness, imageability, familiarity and emotional content were controlled by presenting every word in each voice across all experimental conditions. The cloze probability was calculated for congruent and incongruent target words with a questionnaire completed by 64 individuals who did not participate in the experiment proper. Congruent target words were predicted in 8.4% of the cases while incongruent words were never predicted.

The experiment was performed in an electrically shielded cabin. Participants were seated in a comfortable chair facing a computer screen (1280 x 1024 pt) at a viewing distance of 60 cm. The auditory stimuli were presented through a pair of shielded speakers placed at both sides of the screen. Sound pressure level was the same for all participants, who all confirmed that the loudness of the voice was comfortable. Every participant listened to 240 sentences evenly distributed to four conditions: 60 congruent and 60 incongruent sentences seeing the speakers, and the same proportion seeing the scrambled faces. Each sentence was presented to a given participant only once. Please note that participants were presented only with 240 out of the total 480 sentences because they would also participate in Experiment 2, where they received the other 240 sentences. Sentence assignment was counterbalanced. The experimental session was divided into two blocks of 120 sentences each according to the type of picture (face vs. scrambled face). Congruent and incongruent sentences were mixed in random order. The order of the blocks was counterbalanced across participants and a pause was included both between blocks as well as within each block, after 60 sentences. We designed 14 presentation sets of 240 sentences each to cover all possible combinations between sentences, semantic congruency and visual stimuli. Two participants were assigned to each presentation set, and the presentation of the sentences within each set was randomized. Across the entire experiment with all participants every sentence had been spoken in all 4 voices, but a given participant heard a given sentence only in one of the voices; hence, differences in the results cannot be attributed to particular linguistic characteristics of the sentences or of the voices. Participants were asked to try not to blink while the sentences were presented. Experiment 1 was separated from Experiment 2 by a 10-min break. Each Experiment took 25 minutes. The order of experiments was counterbalanced across participants.
